# Protein Kinase C (PKC) in Neurological Health: Implications for Alzheimer’s Disease and Chronic Alcohol Consumption

**DOI:** 10.3390/brainsci14060554

**Published:** 2024-05-29

**Authors:** Nishtha Singh, Shouvik Kumar Nandy, Anupam Jyoti, Juhi Saxena, Aditi Sharma, Arif Jamal Siddiqui, Lalit Sharma

**Affiliations:** 1 Department of Pharmacology, School of Pharmaceutical Sciences, Shoolini University of Biotechnology, and Management Sciences, Solan 173229, Himachal Pradesh, India; dogranishtha93@gmail.com (N.S.); aditisharma31790@gmail.com (A.S.); 2 School of Pharmacy, Techno India University, Sector-V, Kolkata 700091, West Bengal, India; shouvikknandy@gmail.com; 3 Department of Life Science, Parul Institute of Applied Science, Parul University, Vadodara 391760, Gujarat, India; anupamjyoti03@gmail.com; 4 Department of Biotechnology, Parul Institute of Technology, Parul University, Vadodara 391760, Gujarat, India; jina.saxena@gmail.com; 5 Department of Biology, College of Science, University of Hail, Hail 55476, Saudi Arabia

**Keywords:** protein kinase C (PKC), brain, AD, synaptic plasticity, neurotransmitter regulation, neuronal signalling, neurodegeneration

## Abstract

Protein kinase C (PKC) is a diverse enzyme family crucial for cell signalling in various organs. Its dysregulation is linked to numerous diseases, including cancer, cardiovascular disorders, and neurological problems. In the brain, PKC plays pivotal roles in synaptic plasticity, learning, memory, and neuronal survival. Specifically, PKC’s involvement in Alzheimer’s Disease (AD) pathogenesis is of significant interest. The dysregulation of PKC signalling has been linked to neurological disorders, including AD. This review elucidates PKC’s pivotal role in neurological health, particularly its implications in AD pathogenesis and chronic alcohol addiction. AD, characterised by neurodegeneration, implicates PKC dysregulation in synaptic dysfunction and cognitive decline. Conversely, chronic alcohol consumption elicits neural adaptations intertwined with PKC signalling, exacerbating addictive behaviours. By unravelling PKC’s involvement in these afflictions, potential therapeutic avenues emerge, offering promise for ameliorating their debilitating effects. This review navigates the complex interplay between PKC, AD pathology, and alcohol addiction, illuminating pathways for future neurotherapeutic interventions.

## 1. Introduction

Protein kinase C (PKC) is an enzyme family that plays a crucial role in cell signalling and regulation [1,2]. Cell development, differentiation, proliferation, apoptosis, and immunological responses are all regulated by these enzymes. Because they phosphorylate target proteins on serine and threonine amino acid residues, PKC enzymes are known as serine/threonine kinases [3]. PKC has numerous isoforms that are classified into three types depending on structural features and activation mechanisms: cPKCs (conventional PKCs)—the activation of these isoforms requires calcium and diacylglycerol (DAG) [4]. Calcium serves as a cofactor, while DAG is a lipid-derived molecule that assists in the recruitment of PKC to the cell membrane, where it may be activated. PKC, PKCI, PKCII, and PKC are examples of cPKCs [5,6]. These isoforms are also dependent on DAG for activation but do not require calcium [7]. PKCs are triggered by a variety of external signals, including growth factors, hormones, and neurotransmitters. These signals set off a chain of events that finally lead to the activation of PKCs [8]. PKCs translocate from the cytoplasm to the cell membrane after activation due to interactions with DAG and other lipids [9]. This step is essential for PKC to gain access to its substrates. PKC phosphorylates target proteins on serine and threonine residues once it is localised at the cell membrane [10,11]. PKCs translocate from the cytoplasm to the cell membrane after activation due to interactions with DAG and other lipids. This step is essential for PKC to gain access to its substrates. PKC phosphorylates target proteins on serine and threonine residues once it is localised at the cell membrane [12]. PKC activity dysregulation has been linked to a variety of illnesses, including cancer, diabetes, cardiovascular disease, and neurological problems. As a result, PKC isoforms have emerged as prospective pharmacological targets for influencing certain cellular processes. PKC is only one component of a complicated signalling network within the cell, and its function is closely controlled to ensure correct cellular responses to external stimuli and the maintenance of cellular homeostasis [13,14]. The PKC isoforms and signalling pathways in which PKC is engaged may differ between organs and tissues. PKC controls cardiac muscle contraction, electrical signalling, and blood vessel tone in the heart. PKC activation can affect normal heart function, as well as cardiovascular disorders such as heart failure, arrhythmias, and hypertension [15]. PKC is involved in hormone release and the control of numerous metabolic processes in endocrine glands such as the pancreas and adrenal glands. PKC governs smooth muscle contraction, digestive enzyme production, and cell development in the gastrointestinal system. PKC dysregulation has been linked to illnesses such as irritable bowel syndrome and colorectal cancer [16]. PKC is involved in the activation, proliferation, and differentiation of immune cells. It is required for the proper functioning of numerous immune cells, including T and B cells [17]. PKC regulates smooth muscle contraction in airways and bronchioles in the lungs. It may also have repercussions for asthma and chronic obstructive pulmonary disease (COPD) [18]. PKC has a role in muscular contraction, glucose uptake, and muscle development. It is applicable to illnesses such as muscular dystrophy and muscle wasting syndromes [19,20]. PKC is involved in glucose metabolism, lipid metabolism, and inflammation in the liver. It is applicable to diseases such as non-alcoholic fatty liver disease (NAFLD) and diabetes [21,22]. PKC is important for synaptic plasticity, learning, and memory in the brain. It also influences neuronal survival, development, and neurotransmitter release [23]

PKC’s position in each organ is very complicated and unique to that organ’s activities. PKC activity must be closely controlled to maintain proper cellular function, and PKC dysregulation can lead to a variety of illnesses [24]. However, while PKC is crucial in many organs, the precise activities and results differ depending on the individual PKC isoforms involved, the cellular environment, and the signalling pathways in which it participates. In the intricate landscape of neurological health, the role of protein kinase C (PKC) stands as a pivotal player, orchestrating crucial cellular processes and signalling pathways [23,24]. Within this context, understanding PKC’s intricate involvement holds profound implications for two significant afflictions of the modern age: AD and chronic alcohol addiction. Both conditions exact a heavy toll on individuals and society, manifesting complex neurological disruptions. Exploring the interplay between PKC, AD pathology, and the neurobiological mechanisms underpinning alcohol addiction unveils a compelling narrative of interconnectedness. This review aims to delve into the nuanced relationship between PKC dysregulation, neurodegeneration in Alzheimer’s Disease, and the neural adaptations perpetuating alcohol addiction, shedding light on potential therapeutic avenues at this intricate crossroads of neurological research.

## 2. PKC and Brain

Protein kinase C is a family of enzymes that plays a central role in various cellular processes, including gene expression, cell proliferation, signal transduction, and synaptic plasticity. It is particularly prominent in the brain as it underlies learning and memory processes [25]. PKC regulates various signalling pathways inside neurons, influencing their activity and communication. PKC is related to the regulation of the ability of neuronal connections to change strength [26,27]. This is crucial to activities such as learning and memory. PKC’s role in signalling cascades can vary the synaptic strength, possibly influencing how information is processed and stored in the brain. [28]. PKC’s involvement in synaptic plasticity is directly linked to cogitative behaviour. It is believed that activating PKC isoforms may increase synaptic transmission and contribute to long-term potentiation, a process that strengthens synaptic connections and is thought to support learning and memory formation [29]. PKC signalling dysregulation has been associated with a range of neurological diseases, including AD and other kinds of dementia. PKC activity, for example, can be changed in AD, impacting synaptic plasticity and cognitive function [30]. PKC is also involved in neurotransmitter release, which is how neurons interact with one another. This can influence general brain function and communication across brain areas [31]. PKC has been demonstrated to alter neuronal cell survival and apoptosis. It has the potential to alter the balance between cell survival and death, which is critical for sustaining appropriate brain function [32]. During brain development, PKCs tend to undergo processes such as neuronal migration, axon guidance, and synaptogenesis. These processes are crucial for the proper wiring of the brain’s circuitry. Overall, the relationship between PKC and the brain is complex and multifaceted [33]. PKC functions in a variety of activities that affect neuronal function, communication, and survival. Its relevance in higher cognitive functions is shown by its role in synaptic plasticity and cognitive formation. It is crucially emphasised, however, that the brain is a very sophisticated and interconnected organ, and the precise methods and results of PKC signalling might vary depending on the context and individual PKC isoforms involved [34,35].

## 3. PKC and AD

PKC is engaged in a variety of activities that regulate neuronal function, communication, and survival. Its role in synaptic plasticity and memory formation emphasises its significance in higher cognitive functions. However, it is crucial to emphasise that the brain is a very sophisticated and interconnected organ, and the precise methods and results of PKC signalling might vary depending on the context and individual PKC isoforms involved [36,37]. PKC regulates synaptic plasticity, the process by which synapses increase or diminish in response to brain activity. Synaptic plasticity is a crucial process underpinning the creation of cogitative memory [38]. The two main kinds of neuronal plasticity are long-lasting potentiation (LTP) and long-term depression (LTD), which rely heavily on synaptic plasticity. LTP is characterised by the strengthening of synaptic connections between neurons, whereas LTD is characterised by the weakening of synaptic connections [39,40]. Both LTP and LTD are regarded as biological pathways in the brain that underpin learning and memory storage. PKC can phosphorylate and alter the activity of neurotransmitter receptors like glutamate receptors [41]. Both LTP and LTD are regarded as biological pathways in the brain that underpin learning and memory storage. PKC can phosphorylate and alter the activity of neurotransmitter receptors like glutamate receptors [42]. PKC participates in several intracellular signalling pathways that regulate neuronal function. These pathways can influence dendritic growth and branching (the receiving ends of neurons, neuronal circuit formation, and the overall functional connectivity of the brain) [43,44]. PKC is also involved in the control of presynaptic neurotransmitter release. This can have an impact on neuron communication and synaptic transmission strength [45]. PKC is engaged in processes linked to neuronal survival and plasticity, in addition to its function in memory and learning. It aids the preservation of neuronal health and the adaptability of neurons to changing surroundings [46].

PKC enzymes are involved in a variety of biological activities, including cell signalling, proliferation, differentiation, and synaptic plasticity. PKC enzymes are activated by various signalling molecules, including calcium ions (Ca^2+^), diacylglycerol (DAG), and phosphatidylserine [47]. PKC enzymes play roles in multiple cellular pathways, including those related to neurotransmitter release, synaptic plasticity, and cellular response to stress. PKC signalling dysregulation has been linked to neurodegenerative illnesses such as Alzheimer’s Disease. There are evidence that beta-amyloid can impact PKC activity and vice versa. For instance, beta-amyloid accumulation and its subsequent aggregation can lead to oxidative stress, which can affect PKC signalling pathways [48]. PKC activation, on the other hand, can impact beta-amyloid processing. PKC activation has been demonstrated to impact APP cleavage, potentially rebalancing the amyloidogenic and non-amyloidogenic pathways [49]. PKC activation may potentially affect the activity of BACE1, the enzyme that initiates beta-amyloid formation. The interaction between beta-amyloid processing and PKC signalling is complicated and poorly understood [50]. Researchers continue to investigate these interactions to gain insights into the mechanisms underlying AD and identify potential therapeutic targets. There is a complex interplay between PKC signalling, beta-amyloid, and tau phosphorylation in AD pathogenesis, Beta-amyloid may reduce PKC activity in a biphasic manner. Beta-amyloid supports PKC activity at low doses but inhibits it at larger quantities [51]. This regulation of PKC by beta-amyloid may contribute to both neurotrophic and neurotoxic effects, impacting processes such as memory loss and neuronal death. Beta-amyloid oligomers activate PKC, which governs the distribution and activity of an NMDA receptor’s NR2B component in neuronal plasma membranes. PKC phosphorylates NR2B at Ser1303, which is blocked by the PKC inhibitor Gö6983. Inhibiting PKCδ significantly decreases BACE1 expression, APP processing, and Aβ generation. The overexpression of PKCδ promotes BACE1 expression and Aβ production [52]. This shows that PKCδ has a significant role in aggravating AD aetiology. Beta-amyloid causes pathology-related patterns of tau phosphorylation at several locations. Six proteins involved in diverse post-translational modification pathways, including PKC, are linked to beta-amyloid-induced tau disease. PKC-mediated tau phosphorylation at certain places may enhance tau aggregation and dissemination, leading to the pathogenesis of AD [53]. It means that Beta-amyloid affects PKC activity, which controls BACE1 expression, APP processing, and tau phosphorylation. The physiology of AD is centred on the intricate interaction between PKC signalling, beta-amyloid, and tau pathology [54].

**Figure 1:**
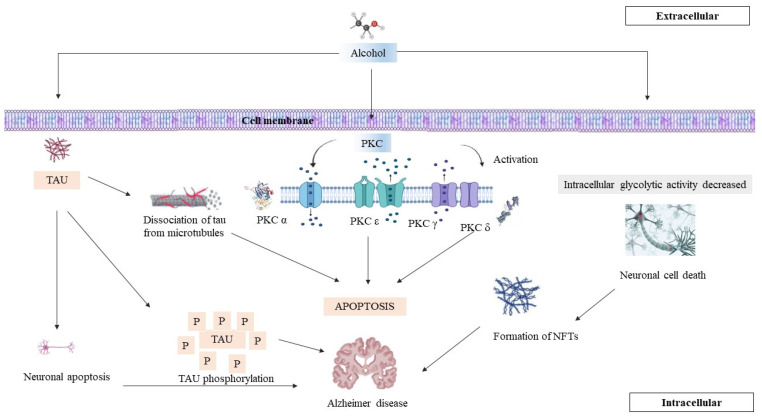
Interactions between tau phosphorylation and PKC. This figure illustrates the relationship between protein kinase C (PKC) activity and tau phosphorylation, highlighting the resulting pathological changes in neuronal structure and function. This results in hyperphosphorylation, unhealthy microtubules, and the increased generation of neurofibril tangles.

PKC has been implicated in the parameter of tau phosphorylation. The relationship between PKC and tau phosphorylation is complex and can have different outcomes depending on the specific isoform of PKC involved, the cellular context, and the phosphorylation sites on tau [55]. Some studies suggest that certain PKC isoforms, particularly PKCδ and PKCε, may contribute to tau phosphorylation. The activation of these PKC isoforms has been linked to increased tau phosphorylation at specific sites. Conversely, other PKC isoforms, such as PKCα and PKCθ, have been reported to have a suppressive effect on tau phosphorylation [56]. It is important to emphasise that the effects of PKC on tau phosphorylation can be context-dependent, and the precise mechanisms involved are still being studied [57,58]. The dysregulation of PKC activity and its impact on tau phosphorylation may play a role in the pathogenesis of neurodegenerative diseases, including AD (Figure 1) [59]. Research in this area continues to evolve, and new findings may shed further light on the complex interactions between tau phosphorylation, PKC, and neurodegenerative processes. The hyperactivation of PKC, induced by different means, has been demonstrated to result in enhanced site-specific tau phosphorylation, as indicated by higher levels of paired helical filament-I (PHF-I) and ALZ-50 immunoreactivity [60]. The downregulation of PKC epsilon reduces PHF-I and ALZ-50 immunoreactivity, confirming its role in controlling the tau phosphorylation processes that create these epitopes. In a similar way, downregulating PKC alpha or epsilon reduces immunoreactivity to phosphate-independent anti-tau antibodies, indicating that they play a role in maintaining tau levels. Significantly, PKC alpha and epsilon appear to have distinct effects on tau regulation, since other PKC isoforms or calcium-dependent kinases are unable to compensate for their downregulation in terms of tau levels or immunoreactivity. The mechanisms of PKC hyperactivation include elevated levels of paired helical filament-I (PHF-I) and ALZ-50 immunoreactivity at particular sites [61]. PKC epsilon downregulation lowers PHF-I and ALZ-50 immunoreactivity, indicating that it modulates the tau phosphorylation processes that generate these epitopes. PKC alpha and epsilon downregulation affects the levels of phosphate-independent anti-tau antibodies, indicating that they play a role in tau steady-state management. PKC alpha and epsilon appear to play separate roles in tau regulation, since other PKC isoforms or calcium-dependent kinases are unable to compensate for reduced tau levels or immunoreactivity indicators [62].

ALKBH5 interacts with diacylglycerol kinase eta (Dgkh) and activates PKC-alpha, resulting in tau hyperphosphorylation in hippocampus neurons. The reduction in PKC-alpha activity reduces tau hyperphosphorylation produced by high glucose levels and/or Dgkh knockdown activation suppresses the activity of glycogen synthase kinase-3 beta. GSK3-beta is a proline-directed protein kinase that phosphorylates tau on serine or threonine residues, followed by a proline residue. Inhibiting GSK3-beta prevents tau protein hyperphosphorylation and reduces beta-amyloid peptide levels. PKC isoforms, namely alpha and epsilon, contribute to tau hyperphosphorylation by directly regulating tau phosphorylation events and indirectly altering the activities of other indicators. The dysregulation of PKC signalling pathways is critical for abnormal tau hyperphosphorylation, which is a hallmark of AD [63].

## 4. PKC and Neuroinflammation

In signalling pathways that regulate cell survival, proliferation, differentiation, and inflammatory responses, PKC isoforms play essential roles. They can either induce or regulate inflammation depending on the PKC isoform [64]. Some PKC isoforms, such as PKCδ and PKCθ, have been associated with pro-inflammatory responses. When activated, they can enhance the formation of inflammatory cytokines and other mediators by immune cells [58]. PKC isoforms activate transcription factors such as NF-κB, regulating inflammation-related gene expression [65]. Conversely, other PKC isoforms, such as PKCα and PKCε, have been linked to anti-inflammatory effects. The activation of these isoforms can suppress the expression of pro-inflammatory cytokines and modulate immune cell activity [66]. The relationship between PKC and neuroinflammation is intricate and context-dependent. In the context of neurodegenerative diseases, including AD, the activation of certain PKC isoforms might be linked to the increased production of inflammatory molecules that contribute to neuroinflammation [67]. This inflammation, in turn, can exacerbate neuronal damage and disease progression. Furthermore, the interaction between PKC and neuroinflammation may contribute to the pathological changes observed in these diseases [68]. For example, beta-amyloid plaques and tau tangles in AD can activate neuroinflammatory responses, and PKC isoforms may alter the production of cytokines and other inflammatory mediators [69,70]. Understanding the interaction between PKC signalling and neuroinflammation is critical for creating possible treatment techniques to control both processes and slow the course of neurodegenerative diseases.

In AD animal models, neuroinflammation is strongly linked to tau pathology. Neu-pro-inflammation, defined by microglial activation and inflammatory responses, plays a significant role in the progression of tau illness, culminating in cognitive impairment and neuronal damage [71]. Tau disease is driven by extended and intensified inflammatory reactions in glial cells and neurons, which exacerbate inflammatory responses. Neuroinflammation may precede β-amyloid and tau abnormalities in late-onset AD, highlighting its importance in disease progression. In AD mice models, glial cell activation and inflammatory factor release are linked to tau hyperphosphorylation and cognitive impairment. Neuroinflammation and PKC isoforms have an influence on tau hyperphosphorylation and beta-amyloid deposition in the brains of AD mice models alpha and epsilon hyperactivation results in enhanced tau phosphorylation at particular sites, as demonstrated by increased paired helical filament-I (PHF-I) and ALZ-50 immunoreactivity levels. Epsilon downregulation lowers PHF-I and ALZ-50 immunoreactivity, indicating that it regulates the tau phosphorylation events that produce these particular epitopes. The inhibition of PKC-alpha activity reduces tau hyperphosphorylation mediated by high glucose and/or Dgkh knockdown in hippocampus neurons [72,73].

PKC activation reduces the action of a crucial tau kinase, glycogen synthase kinase-3 beta (GSK3-beta). The inhibition of GSK3-beta inhibits the hyperphosphorylation of tau protein. Increased PKCδ can enhance neurodegenerative signalling pathways, including neuroinflammation, oxidative stress, and apoptosis, during ageing and neurotoxicity [74,75]. Aβ-induced inflammation is linked to AD brain pathology. Pro-inflammatory transcriptional regulators like NF-κB may play a role in mediating or worsening neurotoxicity caused by Aβ, leading to higher NF-κB p65 activity and BACE1 expression in the postmortem AD brain. Given that NF-κB promoter elements may be located within the BACE1 locus, the upregulation of NF-κB may be connected to increased Aβ production in AD. In a transgenic AD mouse model, inhibiting m-[PKCδ with rottlerin reduces BACE1, Aβ levels, and plaque formation while improving cognitive impairments [68,76].

Restoring PKC ε cytosol-to-cell membrane translocation and activity decreases neurofibrillary tangles and Aβ deposition in transgenic animal models. The dysregulation of various PKC isoforms, notably PKC alpha, epsilon, and delta, is linked to tau hyperphosphorylation and beta-amyloid accumulation in AD. Neuroinflammation caused by PKC signalling pathways and transcriptional regulators, such as NF-κB, exacerbates these clinical symptoms. Modulating PKC activity, particularly PKC epsilon and delta, has potential as a treatment method in AD. The complicated processes by which PKC isoforms regulate neuroinflammation and its consequences for neurological diseases are still being studied [77]. The complicated processes by which PKC isoforms regulate neuroinflammation and its consequences for neurological diseases are still being studied.

## 5. Dual Role of PKC in Neuroprotection and Neurodegeneration

PKC activation has emerged as a double-edged sword in AD research, with some isoforms potentially offering neuroprotection against neurodegeneration [78].

### 5.1. Restoring Ion Channel Function and Reduced Neuronal Excitability

The proper functioning of potassium (K+) channels is essential for neuronal communication. AD is associated with K+ channel dysfunction. Research indicates that PKC activation can restore this function in cells derived from AD patients, potentially improving neuronal signalling [79]. By restoring K+ channel function, PKC activation could improve the way that neurons communicate with each other, potentially mitigating the cognitive decline seen in AD. The dysfunction of K+ channels can lead to excessive neuronal firing, a phenomenon linked to neurodegeneration. Restoring K+ channel function through PKC activation might help to maintain a healthy balance of neuronal activity. By preventing K+ channel dysfunction and neuronal hyperexcitability in healthy brains, K+ channels maintain a proper balance of electrical activity in neurons [80]. In AD, K+ channels malfunction occurs, leading to excessive leakage of potassium ions out of the neuron. This disrupts the neuron’s ability to regulate its electrical activity, resulting in hyperexcitability—excessive firing. Chronic hyperexcitability is linked to neurodegeneration, a hallmark of AD [81].

### 5.2. Maintaining Calcium Homeostasis and Promoting Neuronal Survival

PKC plays a role in regulating calcium levels within neurons. A balanced calcium concentration is crucial for neuronal health. PKC activation might help to maintain this balance, protecting neurons from excitotoxicity, a process linked to AD neurodegeneration [76]. Calcium ions (Ca^2^⁺) act as crucial signalling molecules within neurons. A tightly regulated balance of calcium is essential for proper neuronal function. In AD, this balance is disrupted, leading to an influx of calcium into neurons. This excessive calcium can trigger a cascade of events that damage and eventually kill neurons, contributing to neurodegeneration [54]. The activation of specific PKC isoforms might trigger signalling pathways that enhance neuronal survival and reduce neurodegeneration [82]. A healthy brain relies on a delicate balance between neuronal survival and death. In AD, this balance is tipped towards neurodegeneration, leading to the progressive loss of neurons in specific brain regions [83]. Studies suggest that the activation of specific PKC isoforms might trigger signalling cascades that promote neuronal survival, and PKC activation could activate signalling pathways that suppress apoptosis in neurons [84]. PKC activation could enhance the ability of neurons to cope with cellular stress, a major contributor to neurodegeneration in AD. Some challenges like the overactivation of PKC can also be detrimental. Finding the right balance in PKC activity is critical for achieving neuroprotection and numerous PKC isoforms, each with potentially distinct roles in AD. The activation of some isoforms, like PKCα (with specific mutations), might be beneficial, while others might worsen neurodegeneration [85].

### 5.3. PKC Isoforms That Have Been Implicated in AD Pathology

The human PKC family consists of fifteen diverse isoforms categorised into three main groups based on their activation needs [86]. The first group, conventional PKCs (cPKCs), requires both calcium and diacylglycerol (DAG) for activation. This group includes alpha (α), beta 1 (β1), beta 2 (β2), and gamma (γ) isoforms [87]. The second group, novel PKCs (nPKCs), only needs DAG for activation and does not require calcium. This group includes delta (δ), epsilon (ε), eta (η), and theta (θ) isoforms. Finally, the atypical PKCs (aPKCs) are independent of both calcium and DAG for activation [88]. This group consists of zeta (ζ) and iota/lambda (ι/λ) isoforms. Isoforms potentially contributing to AD are conventional PKCs (cPKCs); conventional PKCs (cPKCs) are a subfamily within the PKC enzyme family [82]. They play a significant role in various cellular processes, but in the context of neurodegeneration, particularly AD, some cPKC isoforms have been implicated in worsening the pathology. PKCα is an isoform that has been implicated in increased Aβ plaque formation, a hallmark of AD. Studies suggest PKCα might influence APP processing, favouring pathways that generate Aβ. PKCα might influence the processing of APP in neurons, favouring pathways that generate Aβ peptides. This could involve directly activating enzymes involved in APP cleavage, triggering signalling cascades that increase Aβ production genes, or affecting APP’s intracellular trafficking. Research into the role of PKC in AD has been advancing rapidly, shedding light on its involvement in the various pathological processes underlying the disease. Recent studies have highlighted the roles of specific PKC isoforms in synaptic dysfunction, a hallmark of early AD [89]. Research suggests that PKCα and PKCε isoforms are involved in regulating synaptic plasticity and neurotransmitter release, and their dysregulation contributes to the synaptic deficits seen in AD [90]. Tau protein hyperphosphorylation is a key event in AD’s pathology [85]. Recent preclinical studies have shown that PKC activation can reduce tau phosphorylation by inhibiting the activity of kinases involved in tau hyperphosphorylation, such as glycogen synthase kinase-3β (GSK-3β) and cyclin-dependent kinase 5 (CDK5) [91]. Neuroinflammation plays a crucial role in the progression of AD. Recent research has demonstrated that PKC activation can modulate microglial activation and reduce the release of pro-inflammatory cytokines, thereby attenuating neuroinflammation in AD mouse models [92]. The disruption of the BBB is a characteristic feature of AD, contributing to neurovascular dysfunction and neuronal damage. Recent studies have shown that PKC activation can enhance BBB integrity by regulating the expression and localisation of tight junction proteins, offering potential therapeutic avenues for AD [93]. Several clinical trials targeting PKC modulation in AD are underway. These trials involve the use of PKC activators or inhibitors to assess their efficacy in improving cognitive function and slowing disease progression. Preliminary results from some early-phase trials suggest potential benefits of PKC modulation in AD, although further research is needed to establish their therapeutic utility [94]. The intricate role of PKC in the pathogenesis of AD highlights its potential as a therapeutic target for the development of novel treatment strategies. However, more extensive preclinical and clinical studies are necessary to fully elucidate the therapeutic benefits and safety profile of targeting PKC in AD [95].

### 5.4. PKC in Regulating Mitochondrial Function and Dynamics in AD

Protein kinase C (PKC) plays a significant role in regulating mitochondrial function and dynamics in AD, impacting oxidative stress, energy metabolism, and neuronal survival [96]. Mitochondria are a major source of reactive oxygen species (ROS), and their dysfunction contributes to oxidative stress in AD [97]. PKC isoforms, particularly PKCδ and PKCε, have been implicated in the regulation of mitochondrial ROS production. PKC activation can modulate the activity of electron transport chain complexes, affecting ROS generation [98]. Additionally, PKC can phosphorylate and regulate antioxidant enzymes such as superoxide dismutase (SOD) and catalase, which play crucial roles in scavenging ROS and maintaining redox balance in neurons [99]. Various isoforms of PKC, including PKCα, PKCδ, and PKCε, are involved in the regulation of oxidative stress in AD. These isoforms exhibit distinct roles in modulating the activity of antioxidant enzymes and ROS-generating enzymes within mitochondria [100]. For instance, PKC activation can enhance the expression and activity of antioxidant enzymes such as SOD and catalase, thus mitigating oxidative stress. Conversely, the PKC-mediated phosphorylation of mitochondrial respiratory chain complexes can influence ROS production, impacting oxidative stress levels in AD neurons [101,102]. Mitochondrial dysfunction is a hallmark of AD pathology, leading to increased ROS production and oxidative damage. PKC signalling pathways modulate mitochondrial function and dynamics, influencing ROS generation within these organelles [103]. Dysregulated PKC activity in AD can disrupt mitochondrial bioenergetics and promote ROS overproduction, exacerbating oxidative stress and neuronal damage. Oxidative stress is closely linked to neuroinflammation, another key feature of AD. PKC signalling pathways intersects with inflammatory pathways, regulating the expression of pro-inflammatory cytokines and inflammatory mediators [104]. By modulating neuroinflammation, PKC can indirectly influence oxidative stress levels in AD brains, highlighting the interconnectedness of these pathological processes. Excessive oxidative stress can trigger neuronal apoptosis, contributing to neurodegeneration in AD [105]. PKC signalling pathways are involved in the regulation of apoptotic pathways within neurons, modulating the balance between pro-survival and pro-apoptotic signals. Dysregulated PKC activity can disrupt this balance, promoting neuronal apoptosis and cell death in AD brains [106]

PKC plays a crucial role in controlling mitochondrial function and dynamics, especially in the context of AD. PKC is a family of serine/threonine kinases that regulate a variety of cellular functions, including mitochondrial biogenesis, dynamics, and apoptosis. In Alzheimer’s dementia, abnormal PKC signalling has been related to mitochondrial dysfunction, which is a hallmark of the disease [96]. PKC regulates mitochondrial dynamics by phosphorylating essential proteins involved in mitochondrial fission and fusion, which are critical for maintaining mitochondrial integrity and function. For example, PKC has been found to phosphorylate Drp1, a protein that promotes mitochondrial fission, resulting in excessive mitochondrial fragmentation, which is typically observed in AD. PKC also regulates mitochondrial activity by influencing the electron transport chain and ATP generation, both of which are critical for neuronal energy demands. PKC activity in AD is frequently dysregulated, resulting in decreased mitochondrial bioenergetics and increased oxidative stress. This imbalance adds to the buildup of beta-amyloid and tau proteins, which worsens mitochondrial damage and neuronal cell death. As a result, targeting PKC pathways might be a promising therapeutic method for restoring mitochondrial function and reducing neurodegeneration in AD [107,108]

Mitochondria are central to cellular energy production through oxidative phosphorylation. PKC signalling influences mitochondrial function by regulating key enzymes involved in energy metabolism, such as pyruvate dehydrogenase (PDH) and cytochrome c oxidase (Complex IV) [109]. The dysregulation of PKC activity in AD can impair mitochondrial bioenergetics, leading to decreased ATP production and metabolic deficits in neurons. Moreover, the PKC-mediated phosphorylation of mitochondrial proteins involved in energy metabolism can impact their activity and contribute to mitochondrial dysfunction in AD [110]. AD is associated with significant alterations in energy metabolism, primarily affecting glucose utilisation and mitochondrial function. One of the earliest and most consistent metabolic abnormalities observed in AD is glucose hypometabolism, particularly in regions of the brain involved in memory and cognition, such as the hippocampus and temporal cortex [50]. Positron emission tomography (PET) studies have shown reduced glucose uptake and metabolism in these brain regions, even in early stages of the disease. Glucose hypometabolism is thought to precede the onset of clinical symptoms and may contribute to synaptic dysfunction and neuronal loss in AD [54]. Glucose is the primary energy substrate utilised by neurons for ATP production through glycolysis and oxidative phosphorylation [111]. In AD, the dysregulation of key enzymes involved in glycolysis, such as hexokinase and pyruvate kinase, impairs glucose metabolism and reduces ATP production. This bioenergetic deficit compromise’s neuronal function and synaptic transmission, contributing to cognitive impairment in AD [112].

Mitochondria play a central role in cellular energy production through oxidative phosphorylation. Mitochondrial dysfunction is a hallmark feature of AD pathology, characterised by impaired mitochondrial respiration through decreased ATP synthesis and increased production of reactive oxygen species (ROS) [113]. Dysfunctional mitochondria contribute to neuronal damage and synaptic loss in AD brains, further exacerbating energy deficits. Mitochondrial dysfunction is a prominent feature of AD pathology, contributing to neuronal damage, synaptic loss, and cognitive decline [114]. Mitochondria are the primary organelles responsible for producing cellular energy in the form of ATP through oxidative phosphorylation [115]. In AD, there is evidence of impaired mitochondrial respiration, characterised by decreased activity of respiratory chain complexes and reduced ATP synthesis. This bioenergetic deficit compromise’s neuronal function and synaptic transmission, contributing to cognitive impairment [116]. Dysfunctional mitochondria are a major source of reactive oxygen species (ROS) in AD brains [117]. Excessive ROS production overwhelms antioxidant defences, leading to oxidative damage to lipids, proteins, and nucleic acids. Oxidative stress further exacerbates mitochondrial dysfunction, creating a vicious cycle of cellular damage and neuronal loss [118]. The proper regulation of mitochondrial dynamics, including fusion, fission, and mitophagy, is crucial for maintaining mitochondrial quality control and cellular homeostasis. In AD, aberrant mitochondrial dynamics result in mitochondrial fragmentation, impaired trafficking, and the accumulation of damaged mitochondria. Disrupted mitochondrial dynamics contribute to neuronal dysfunction and synaptic loss in AD brains [119].

Mitochondria possess their own DNA (mtDNA), susceptible to oxidative damage and mutations [120]. In AD, mtDNA damage accumulates due to increased oxidative stress and impaired DNA repair mechanisms. Mutations in mtDNA disrupt mitochondrial function and contribute to neuronal dysfunction and neurodegeneration [121]. Mitochondria play a crucial role in maintaining calcium homeostasis within neurons [122]. In AD, dysregulated calcium signalling leads to mitochondrial calcium overload, triggering mitochondrial permeability transition pore (mPTP) opening and the subsequent release of pro-apoptotic factors. Calcium-induced mitochondrial dysfunction exacerbates neuronal death and contributes to disease progression [123]. Pathological hallmarks of AD, including Aβ plaques and tau neurofibrillary tangles, directly impact mitochondrial function. Aβ accumulates within mitochondria, disrupting electron transport chain complexes and impairing ATP production. Tau protein interacts with mitochondrial proteins, leading to mitochondrial fragmentation and dysfunction [124,125]. Energy-sensing pathways, such as AMP-activated protein kinase (AMPK) and mammalian target of rapamycin (mTOR) signalling, are dysregulated in AD [126]. AMPK activation, in response to energy depletion, promotes cellular survival mechanisms such as autophagy and mitochondrial biogenesis. In AD, AMPK activity is impaired, compromising cellular stress responses and exacerbating energy deficits. Dysregulated mTOR signalling, which regulates protein synthesis and cell growth in response to energy availability, also contributes to synaptic dysfunction and neurodegeneration in AD. AD alters energy-sensing pathways contribute to the metabolic dysregulation observed in affected neurons [127].

AMPK is a central regulator of cellular energy homeostasis, activated in response to a decrease in cellular ATP levels or an increase in AMP levels. The activation of AMPK promotes catabolic processes, such as glucose uptake, fatty acid oxidation, and autophagy, while inhibiting anabolic pathways such as protein synthesis and lipogenesis. In AD, the dysregulation of AMPK signalling has been observed, with studies reporting reduced AMPK activity in affected brain regions. Impaired AMPK signalling compromises cellular stress responses, exacerbating energy deficits and neuronal damage in AD [115,128]. mTOR is a key regulator of cell growth, proliferation, and metabolism, integrating signals from growth factors, nutrients, and energy status. The activation of mTOR promotes protein synthesis and cell growth while inhibiting autophagy and mitochondrial biogenesis [129]. Dysregulated mTOR signalling has been implicated in AD pathology, with studies reporting increased mTOR activity in AD brains. The hyperactivation of mTOR contributes to synaptic dysfunction, neuroinflammation, and neuronal loss in AD, exacerbating disease progression [130]. Sirtuins are a family of NAD+-dependent deacetylases involved in regulating cellular metabolism, stress responses, and longevity [131]. Sirtuins play a crucial role in maintaining mitochondrial function, DNA repair, and protein homeostasis. The dysregulation of sirtuin signalling has been implicated in AD pathology, with studies reporting decreased sirtuin expression and activity in AD brains. Impaired sirtuin function compromises mitochondrial biogenesis, antioxidant defences, and DNA repair mechanisms, exacerbating neuronal damage and cognitive decline in AD [132,133]. Insulin signalling plays a crucial role in regulating glucose metabolism, synaptic function, and neuronal survival in the brain [134,135]. Insulin resistance, characterised by impaired insulin signalling and reduced responsiveness to insulin, has been implicated in AD pathology. Dysregulated insulin signalling disrupts glucose uptake, impairs synaptic plasticity, and promotes neuroinflammation in AD brains. Insulin resistance exacerbates energy deficits and neuronal dysfunction, contributing to disease progression [136].

NRF1 (Nuclear Respiratory Factor 1) is a transcription factor that regulates the expression of nuclear genes encoding mitochondrial proteins involved in oxidative phosphorylation, mitochondrial biogenesis, and antioxidant defences [137]. The dysregulation of NRF1 signalling has been observed in AD, with studies reporting decreased NRF1 expression and activity in affected brain regions. Impaired NRF1 function compromise’s mitochondrial function, exacerbating energy deficits and oxidative stress in AD neurons [138,139]. In addition to glucose, neurons can utilise alternative energy substrates such as ketone bodies and fatty acids for ATP production [140]. Ketone bodies, derived from fatty acid oxidation in the liver, serve as an alternative energy source during periods of glucose scarcity or metabolic stress. Ketone metabolism is impaired in AD, contributing to energy deficits and neuronal dysfunction [141]. Therapeutic strategies aimed at enhancing ketone utilisation, such as ketogenic diets or ketone esters, have shown promise in preclinical studies and clinical trials for AD [142]. PKC signalling also plays a role in the regulation of mitochondrial dynamics, including fusion, fission, and mitophagy. Imbalance in mitochondrial dynamics, characterised by excessive fission and inadequate fusion, is observed in AD, and contributes to mitochondrial fragmentation and dysfunction. PKC isoforms such as PKCδ and PKCβ have been implicated in the phosphorylation of mitochondrial fission and fusion proteins, influencing their activity and subcellular localisation. Dysregulated PKC signalling can disrupt mitochondrial dynamics, impairing mitochondrial quality control mechanisms and promoting neuronal damage in AD [143]. Mitochondria are intimately involved in regulating apoptotic pathways, and PKC signalling influences neuronal survival by modulating mitochondrial apoptotic signalling. PKC activation can regulate the expression and activity of anti-apoptotic proteins such as Bcl-2 and Bcl-xL, as well as pro-apoptotic proteins like Bax and Bad, thereby modulating mitochondrial membrane permeability and cytochrome c release. Dysregulated PKC signalling in AD can tilt the balance towards apoptotic cell death, contributing to neuronal loss and cognitive decline [130].

The relationship between tau, beta-amyloid (Aβ) deposition, and inflammation-dependent responses in neurodegeneration is complex, including many pathways such as AMPK and Nrf2. AD is characterised by extracellular Aβ plaques and intracellular tau tangles [144]. These abnormal protein accumulations cause neuroinflammatory responses, largely through the activation of microglia and astrocytes, resulting in a persistent inflammatory state that worsens neuronal damage and disease development. AMPK and Nrf2 regulate cellular energy balance and oxidative stress responses, respectively, and modulate the effects of tau and Aβ. AMPK activity promotes energy balance and mitochondrial function, but dysregulation leads to increased tau phosphorylation and Aβ generation. In contrast, Nrf2 is a transcription factor that activates antioxidant response elements to counteract oxidative stress. Nrf2 pathways are often disrupted in Alzheimer’s patients, resulting in inadequate antioxidant defences and worsening inflammation and neurodegeneration. Increasing Nrf2 activity can reduce oxidative damage, inflammation, tau pathology, and Aβ buildup. Modulating the AMPK and Nrf2 pathways can disrupt the loop of inflammation, tau, and Aβ pathology, potentially slowing down the disease [145].

The relationship between alcohol neurotoxicity, AD, and inflammation is complicated, including several processes. Chronic alcohol intake can cause neurotoxicity, inflammation, and oxidative stress in the brain, all of which can contribute to brain damage and cognitive decline. Alcohol-induced neuroinflammation activates immune cells in the brain, such as microglia, resulting in inflammatory responses that may contribute to neurodegeneration. This inflammatory response can also disrupt brain–body communication networks, potentially resulting in neurotoxicity and cognitive impairment [146,147].

Chronic alcohol misuse and the ensuing neuroinflammation can worsen the degenerative processes linked with AD. Alcohol-related brain inflammation and oxidative stress might contribute to the development or progression of neurodegenerative diseases such as Alzheimer’s Disease. The inflammatory reactions generated by alcohol intake may interact with the underlying processes of AD, thereby hastening cognitive loss and neurodegeneration. Chronic alcohol misuse and the ensuing neuroinflammation can worsen the degenerative processes linked to AD. According to research, alcohol-related brain inflammation and oxidative stress might contribute to the development or progression of neurodegenerative diseases such as Alzheimer’s Disease. The inflammatory reactions generated by alcohol intake may interact with the underlying processes of AD, thereby hastening cognitive loss and neurodegeneration [148].

### 5.5. Involvement of PKC Signalling Pathways in Synaptic Dysfunction

PKC signalling pathways play a crucial role in synaptic dysfunction observed in AD, affecting neurotransmitter release, synaptic plasticity, and dendritic spine morphology [149]. PKC signalling regulates neurotransmitter release by modulating the activity of presynaptic proteins involved in synaptic vesicle exocytosis and neurotransmitter release. In AD, dysregulated PKC activity disrupts neurotransmitter release mechanisms, leading to impaired synaptic transmission [150]. For example, PKC phosphorylates synapsin proteins, which regulate synaptic vesicle trafficking and neurotransmitter release. Dysregulated PKC-mediated phosphorylation of synapsins in AD alters synaptic vesicle dynamics, impairing neurotransmitter release and synaptic function. PKC signalling pathways play a crucial role in regulating synaptic plasticity, including LTP and LTD, which are cellular mechanisms underlying learning and memory [135]. In AD, dysregulated PKC activity disrupts synaptic plasticity processes, impairing the ability of synapses to undergo activity-dependent changes in strength. For example, PKC phosphorylates NMDA receptors and AMPA receptors, which are critical for synaptic plasticity. The dysregulated PKC-mediated phosphorylation of these receptors in AD alters their function, impairing synaptic plasticity and cognitive function [151]. Dendritic spines are small protrusions on dendrites that form synapses with axons and play a crucial role in synaptic transmission and plasticity. PKC signalling pathways regulate dendritic spine morphology by modulating the actin cytoskeleton and spine dynamics. In AD, dysregulated PKC activity disrupts dendritic spine morphology, leading to alterations in spine density, size, and stability. For example, PKC phosphorylates actin-binding proteins such as MARCKS and profilin, which regulate actin dynamics in dendritic spines. The dysregulated PKC-mediated phosphorylation of these proteins in AD alters actin remodelling, leading to aberrant dendritic spine morphology and synaptic dysfunction [152].

## 6. Chronic Alcohol Consumption and Brain

**Figure 2:**
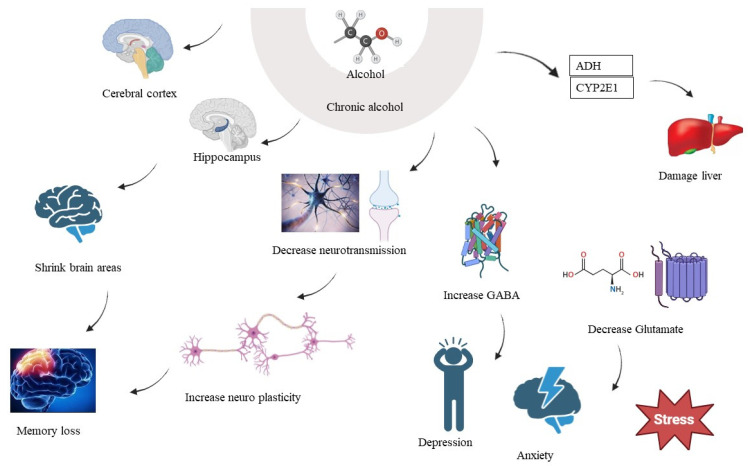
Alcohol and the brain. This figure illustrates the metabolic processes of alcohol in the liver and its subsequent impact on the brain, highlighting the role of oxidative stress and PKC activation. Alcohol is primarily metabolised in the liver by enzymes like alcohol dehydrogenase (ADH) and cytochrome P450 2E1 (CYP2E1) [68,76]. The metabolism of alcohol generates reactive oxygen species (ROS) and can lead to oxidative stress. Oxidative stress can activate PKC isoforms, leading to altered signalling pathways and potentially contributing to liver damage and inflammation.

Alcohol is a neurotoxic drug, which means that it can harm nerve cells in the brain. Long-term and excessive alcohol intake can cause neuronal cell death, especially in brain areas related to learning, memory, and cognitive functioning. Chronic alcohol consumption can cause brain atrophy or a decrease in brain volume [153,154]. The cerebral cortex and the hippocampus are two brain areas that can decrease because of this shrinking. The hippocampus is essential for the formation of new memories, and its atrophy can result in memory problems [155,156]. Long-term alcohol usage can cause cognitive deficiencies such as attention, concentration, decision-making, and problem-solving difficulties [157]. These cognitive impairments can be attributed to both the direct neurotoxic effects of alcohol and the brain damage caused by alcohol-related conditions [158]. This is a severe neurological disorder often associated with chronic alcoholism. It results from a deficiency of thiamine (vitamin B1) due to poor nutrition and impaired absorption [159,160]. Wernicke-Korsakoff syndrome can lead to confusion, memory problems, and difficulty with coordination. Alcohol affects the balance of neurotransmitters in the brain, such as gamma-aminobutyric acid (GABA) and glutamate [161,162]. Chronic alcohol use can lead to an increase in GABA activity (which has sedative effects) and a decrease in glutamate activity (which is excitatory and important for cognitive functions). Chronic alcohol use can disrupt the functioning of brain circuits involved in reward, motivation, and decision-making [146]. This disruption can contribute to addiction and make it difficult for individuals to control their alcohol consumption. Chronic alcohol usage is linked to an increased risk of acquiring mental illnesses, such as depression and anxiety. These conditions can further exacerbate the negative impact of alcohol on brain function. In severe cases, chronic alcohol use can lead to alcohol-induced dementia or alcohol-related brain damage. This condition involves profound cognitive impairment and memory deficits that can be irreversible, which is also known as alcohol-related dementia [163]. It is important to note that the brain has some capacity for recovery and repair if alcohol consumption is stopped. However, some of the damage caused by chronic alcohol use may be permanent, especially in cases of long-term heavy drinking (Figure 2). Alcohol is largely metabolised in the liver by enzymes such as ADH and CYP2E1. Alcohol metabolism creates reactive oxygen species (ROS), which can result in oxidative damage [104,164].

## 7. PKC and Chronic Alcohol Consumption

**Figure 3:**
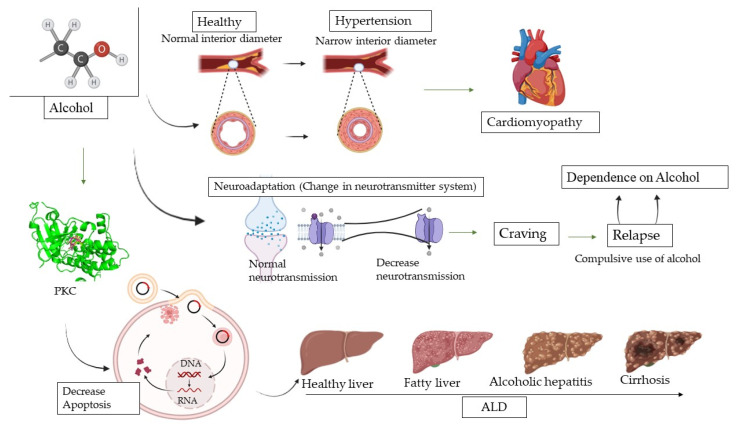
PKC and alcohol consumption. This figure illustrates the effects of alcohol consumption on protein kinase C (PKC) activity and production, as well as its implications for alcoholic liver disease (ALD). Alcohol enhances interaction of the activator with PKc; this interaction occurs within channels for the activation of ethanol. PKC modulates the downregulation; in chronic alcohol consumption, there is a decrease in the production of PKC. Alcoholic liver disease, ALD; protein kinase C, PKC.

Chronic alcohol consumption can lead to alcoholic liver disease (ALD), which encompasses a range of liver conditions, including fatty liver disease, alcoholic hepatitis, fibrosis, and cirrhosis [165]. PKC has been implicated in the development and progression of ALD. It can influence the inflammatory response, apoptosis, and fibrosis in liver cells. Repeated exposure to alcohol can lead to the development of tolerance, where the body becomes less responsive to the effects of alcohol. PKC signalling pathways may be involved in the cellular mechanisms underlying the development of alcohol tolerance. PKC also plays a role in cardiovascular function. Chronic alcohol consumption can lead to cardiovascular issues such as hypertension and cardiomyopathy (Figure 3) [166]. Chronic alcohol consumption can result in neuroadaptations, which are changes in neuronal signalling pathways in response to alcohol exposure. These adaptations can contribute to alcohol cravings, withdrawal symptoms, and relapse. PKC has been implicated in some of these neuroadaptations [167]. PKC is also abundant in the brain and involved in various neuronal functions, including synaptic plasticity and neurotransmitter release. Chronic alcohol use can affect PKC activity in the brain, contributing to neuroadaptive changes and potentially leading to alcohol tolerance and dependence [142].

## 8. PKC, AD, and Chronic Alcohol Consumption

**Figure 4:**
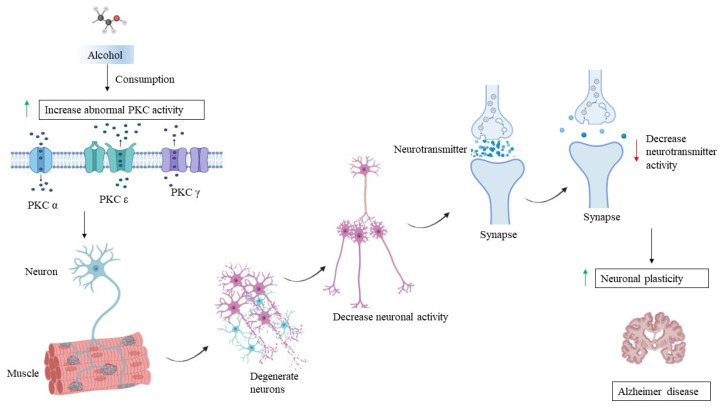
The interplay between protein kinase C (PKC), chronic alcohol consumption, and AD. This figure illustrates the complex relationships between protein kinase C (PKC) activity, chronic alcohol consumption, and the development and progression of Alzheimer’s Disease (AD). Red arrow—decrease. Green arrow—increase.

There is evidence to suggest a connection between PKC, long-term alcohol consumption, and AD. However, the exact nature of this relationship is not yet fully understood [153]. PKC plays a role in processes that are important for synaptic plasticity, learning, memory, and neuronal survival. These functions are crucial for maintaining health [168]. Abnormal PKC activity has been observed in the brains of individuals with AD. Such abnormal activity could contribute to the dysfunction, cognitive decline, and neurodegeneration seen in AD [169]. Chronic alcohol consumption can result in changes in PKC activity and expression in the brain. Factors like alcohol-induced stress, inflammation, and alterations in signalling pathways may affect the functioning of PKC [170]. These changes might contribute to neuronal dysfunction and cognitive deficits associated with chronic alcohol consumption. Some studies have suggested that chronic alcohol consumption might be a risk factor for the development of AD [171]. Excessive alcohol use can lead to oxidative stress, inflammation, and disruptions in brain structure and function, which are also seen in AD. These shared mechanisms raise the possibility that chronic alcohol consumption could contribute to the development or progression of AD [172]. Both chronic alcohol consumption and AD are associated with neuroinflammation, which involves the activation of immune cells and the release of inflammatory molecules in the brain (Figure 4). PKC signalling can modulate inflammation. The dysregulation of PKC-mediated inflammation might contribute to the neuroinflammatory response in both conditions. Both chronic alcohol consumption and AD are linked to synaptic dysfunction and memory impairment. PKC is involved in the regulation of synaptic plasticity and memory formation. Changes in PKC activity could affect these processes in both scenarios [173]. It is important to note that the interaction between PKC, chronic alcohol consumption, and AD is complex and not fully understood. Other factors, such as genetics, age, the duration of alcohol consumption, and overall health, also play a role in determining the outcome. Given the potential role of PKC in both chronic alcohol-related brain dysfunction and AD, there is interest in exploring PKC as a therapeutic target. Modulating PKC activity could potentially offer a strategy to mitigate cognitive impairments associated with these conditions [174]. However, developing such therapies requires a deep understanding of PKC’s specific roles and interactions in these contexts. Alcohol disrupts the gut barrier, allowing endotoxins such as lipopolysaccharides (LPS) to enter the bloodstream and pass the blood–brain barrier (BBB), activating microglia. Alcohol metabolism generates ROS, which damage neuronal cells and activate microglia. Chronic alcohol use alters the neuroimmune environment, inducing pro-inflammatory gene expression in microglia. The effects of microglial overactivation on alcohol-induced neurodegeneration are as follows: Persistent microglial activation by alcohol worsens neuroinflammation and leads to neurodegenerative processes. Microglia produce pro-inflammatory cytokines and ROS, impairing synapse development and function [175], though it is important to mention that while research has provided insights into the possible connections between PKC, chronic alcohol consumption, and AD, this field of study is still evolving, and there is ongoing research being performed to help us to better understand the underlying mechanisms and potential therapeutic avenues.

## 9. Conclusions

In conclusion, the multifaceted role of PKC in neurological health emerges as a central theme in our understanding of brain function and dysfunction. Through this review, we have traversed the intricate landscape of PKC’s involvement in AD pathogenesis and chronic alcohol addiction, shedding light on the interconnected mechanisms driving these debilitating conditions. In Alzheimer’s Disease, PKC dysregulation intertwines with synaptic dysfunction and cognitive decline, contributing to the neurodegenerative cascade characteristic of the disease. Similarly, chronic alcohol consumption elicits neural adaptations intertwined with PKC signalling, exacerbating addictive behaviours and perpetuating a cycle of dependency. By elucidating these relationships, we unveil promising avenues for therapeutic intervention. Targeting PKC signalling pathways holds potential for mitigating the progression of Alzheimer’s Disease and attenuating the neurobiological underpinnings of alcohol addiction. Moreover, the nuanced understanding of PKC’s role in neurological health paves the way for the development of novel treatments that address the intricate interplay between molecular mechanisms and clinical manifestations. As we navigate this complex terrain, interdisciplinary collaboration and innovative research methodologies will be paramount in advancing our knowledge and translating discoveries into tangible benefits for patients afflicted by AD, alcohol addiction, and related neurological disorders. Through concerted efforts, we can harness the therapeutic potential of PKC modulation, offering hope for improved outcomes and enhanced quality of life for those affected by these challenging conditions. PKC regulation has the potential to cure neurodegenerative and addictive illnesses, which is both intriguing and problematic. The intricacy of PKC signalling pathways necessitates a delicate balance between effectiveness and safety. As research advances, it is critical to retain a patient-centred approach, ensuring that the advantages of new medicines outweigh the hazards. Interdisciplinary collaboration will be critical for transferring fundamental research into therapeutic applications. We can speed up the discovery and development of effective therapies by promoting collaborations across several scientific disciplines. Furthermore, working with patients and their families throughout the study process ensures that the therapeutic options created are in line with their needs and expectations. Finally, through collaborative efforts and innovative research methodologies, we can unlock the therapeutic potential of PKC modulation, providing hope for better outcomes and a higher quality of life for those suffering from AD, alcoholism, and other neurological disorders. The trip ahead is difficult, but with perseverance and teamwork, significant progress is possible. The diversity of PKC isoforms and their functions in various neurological processes imply that personalised medicine techniques might improve treatment effectiveness. Interventions tailored to unique PKC isoform dysregulation in individual individuals may enhance results. To further our knowledge of PKC’s involvement in neurological health, we will need to work together across disciplines, combining insights from molecular biology, neurology, pharmacology, and clinical research. This comprehensive approach can encourage the development of novel ways for successfully modulating PKC activity. Identifying accurate biomarkers for PKC activity might help with the early detection and tracking of treatment outcomes in AD and alcoholism. Such biomarkers would be extremely useful in clinical trials and for tailoring treatment interventions. Ongoing research into small chemical inhibitors, modulators, and gene therapy techniques for PKC pathways shows promise. Developing these therapeutic modes could offer new hope for patients with AD, alcohol addiction, and, potentially, other PKC-related neurological disorders.
